# Estimating typhoid incidence from community-based serosurveys: a multicohort study

**DOI:** 10.1016/S2666-5247(22)00114-8

**Published:** 2022-08

**Authors:** Kristen Aiemjoy, Jessica C Seidman, Senjuti Saha, Sira Jam Munira, Mohammad Saiful Islam Sajib, Syed Muktadir Al Sium, Anik Sarkar, Nusrat Alam, Farha Nusrat Zahan, Md Shakiul Kabir, Dipesh Tamrakar, Krista Vaidya, Rajeev Shrestha, Jivan Shakya, Nishan Katuwal, Sony Shrestha, Mohammad Tahir Yousafzai, Junaid Iqbal, Irum Fatima Dehraj, Yasmin Ladak, Noshi Maria, Mehreen Adnan, Sadaf Pervaiz, Alice S Carter, Ashley T Longley, Clare Fraser, Edward T Ryan, Ariana Nodoushani, Alessio Fasano, Maureen M Leonard, Victoria Kenyon, Isaac I Bogoch, Hyon Jin Jeon, Andrea Haselbeck, Se Eun Park, Raphaël M Zellweger, Florian Marks, Ellis Owusu-Dabo, Yaw Adu-Sarkodie, Michael Owusu, Peter Teunis, Stephen P Luby, Denise O Garrett, Farah Naz Qamar, Samir K Saha, Richelle C Charles, Jason R Andrews

**Affiliations:** a Division of Infectious Diseases and Geographic Medicine, Stanford University School of Medicine, Stanford, CA, USA; b Division of Epidemiology, Department of Public Health Sciences, University of California Davis School of Medicine, Davis, CA, USA; c Sabin Vaccine Institute, Washington, DC, USA; d Child Health Research Foundation, Dhaka, Bangladesh; e Dhulikhel Hospital, Kathmandu University Hospital, Dhulikhel, Nepal; f Department of Paediatrics and Child Health, Aga Khan University, Karachi, Pakistan; g Global Immunization Division, Centers for Disease Control and Prevention, Atlanta, GA, USA; h Division of Infectious Diseases, Massachusetts General Hospital, Boston, MA, USA; i Center for Celiac Research and Treatment, MassGeneral Hospital for Children, Boston, MA, USA; j Division of Pediatric Gastroenterology and Nutrition, MassGeneral Hospital for Children, Boston, MA, USA; k Harvard Medical School, Harvard University, Boston, MA, USA; l Department of Immunology and Infectious Diseases, Harvard TH Chan School of Public Health, Harvard University, Boston, MA, USA; m Department of Medicine, University of Toronto, Toronto, ON, Canada; n International Vaccine Institute, Seoul, South Korea; o Cambridge Institute of Therapeutic Immunology and Infectious Disease, University of Cambridge School of Clinical Medicine, Cambridge Biomedical Campus, Cambridge, UK; p Department of Microbiology and Parasitology, University of Antananarivo, Antananarivo, Madagascar; q Heidelberg Institute of Global Health, University of Heidelberg, Heidelberg, Germany; r School of Medical Sciences, Kwame Nkrumah University for Science and Technology, Kumasi, Ghana; s Center for Global Safe Water, Sanitation and Hygiene, Hubert Department of Global Health, Rollins School of Public Health, Emory University, Atlanta, GA, USA; t Bangladesh Council of Scientific and Industrial Research, Dhaka, Bangladesh

## Abstract

**Background:**

The incidence of enteric fever, an invasive bacterial infection caused by typhoidal *Salmonellae* (*Salmonella enterica* serovars Typhi and Paratyphi), is largely unknown in regions without blood culture surveillance. The aim of this study was to evaluate whether new diagnostic serological markers for typhoidal *Salmonella* can reliably estimate population-level incidence.

**Methods:**

We collected longitudinal blood samples from patients with blood culture-confirmed enteric fever enrolled from surveillance studies in Bangladesh, Nepal, Pakistan, and Ghana between 2016 and 2021 and conducted cross-sectional serosurveys in the catchment areas of each surveillance site. We used ELISAs to measure quantitative IgA and IgG antibody responses to hemolysin E and *S* Typhi lipopolysaccharide. We used Bayesian hierarchical models to fit two-phase power-function decay models to the longitudinal antibody responses among enteric fever cases and used the joint distributions of the peak antibody titres and decay rate to estimate population-level incidence rates from cross-sectional serosurveys.

**Findings:**

The longitudinal antibody kinetics for all antigen-isotypes were similar across countries and did not vary by clinical severity. The seroincidence of typhoidal *Salmonella* infection among children younger than 5 years ranged between 58·5 per 100 person-years (95% CI 42·1–81·4) in Dhaka, Bangladesh, to 6·6 per 100 person-years (4·3–9·9) in Kavrepalanchok, Nepal, and followed the same rank order as clinical incidence estimates.

**Interpretation:**

The approach described here has the potential to expand the geographical scope of typhoidal *Salmonella* surveillance and generate incidence estimates that are comparable across geographical regions and time.

**Funding:**

Bill & Melinda Gates Foundation.

**Translations:**

For the Nepali, Bengali and Urdu translations of the abstract see Supplementary Materials section.

## Introduction

Enteric fever, an invasive infection caused by *Salmonella enterica* subspecies *enterica* serovars Typhi and Paratyphi A, B, or C, is a substantial cause of preventable morbidity and mortality in low-income and middle-income countries (LMICs).^1^ Enteric fever incidence is typically ascertained using clinical surveillance, whereby blood culture-positive cases are tallied and reported relative to a catchment area population. Blood culture requires considerable laboratory infrastructure, which is not widely accessible in many LMICs. Another limitation is that only the data on individuals seeking care at surveillance sites are captured, whereas many patients receive treatment from health-care providers outside the reach of traditional surveillance systems. Even when available, the estimated sensitivity of blood culture is only 60%.^2^ Consequently, blood culture surveillance covers a small proportion of at-risk populations and underestimates the global burden of disease. Population-based serological surveillance (serosurveillance) can estimate infection transmission in settings without facility-based surveillance. Cross-sectional serosurveillance has been helpful for rapidly characterising transmission of COVID-19, pertussis, dengue, and other diseases.^3^, ^4^, ^5^, ^6^, ^7^ However, serosurveillance for typhoidal *Salmonella* has been limited by the scarcity of sensitive and specific serological markers. Serological responses to the most widely used antigen to date, virulence (Vi) capsular polysaccharide, have poor diagnostic performance during acute infection and cannot distinguish between natural infection and Vi-based vaccination.^8^ Immune responses to hemolysin E (HlyE) and *S* Typhi lipopolysaccharide demonstrate diagnostic accuracy for acute enteric fever^9^, ^10^, ^11^ but have not been evaluated as tools to measure population-level seroincidence. HlyE, a cytotoxic pore-forming toxin that invades epithelial cells,^12^, ^13^, ^14^ is present in *S* Typhi and *S* Paratyphi A but rarely found in other serovars.^15^ Lipopolysaccharide is a major component of the outer membrane of Gram-negative organisms and a potent inducer of innate immunity.^16^

**Research in context**

**Evidence before this study**

Previous studies have identified serologic responses to two antigens (Hemolysin E [HlyE] and *Salmonella* lipopolysaccharide) as promising diagnostic markers of acute typhoidal *Salmonella* infection. We reviewed the evidence for seroepidemiology tools for enteric fever available as of November 01, 2021, by searching the National Library of Medicine article database and medRxiv for preprint publications, published in English, using the terms “enteric fever”, “typhoid fever”, “*Salmonella* Typhi”, “*Salmonella* Paratyphi”, “typhoidal *Salmonella*”, “Hemolysin E”, “*Salmonella* lipopolysaccharide”, “seroconversion”, “serosurveillance”, “seroepidemiology”, “seroprevalence” and “seropositivity”. We found no studies using HlyE or lipopolysaccharide as markers to measure the incidence or prevalence of enteric fever in a population. Anti-Vi IgG responses were used as a marker of population seroprevalence in cross-sectional studies conducted in South Africa, Fiji, and Nepal, but were not used to calculate population-based incidence estimates.

**Added value of this study**

We developed and validated a method to estimate typhoidal *Salmonella* incidence in cross-sectional population samples using antibody responses measured from dried blood spots. Using longitudinal dried blood spots collected from over 1400 blood culture-confirmed patients in four countries, we modelled the longitudinal dynamics of antibody responses for up to 2 years following infection, accounting for heterogeneity in antibody responses and age dependence. We found that longitudinal antibody responses were highly consistent across four countries on two continents and did not differ by clinical severity. We then used these antibody kinetic parameters to estimate incidence in population-based samples in six communities across the four countries, whereby concomitant population-based incidence was measured using blood cultures. Seroincidence estimates were much higher than blood-culture-based case estimates across all six sites, suggestive of a high incidence of asymptomatic or unrecognised infections. Still, the rank order of seroincidence and culture-based incidence rates were the same, with the highest rates in Bangladesh and lowest in Ghana.

**Implications of all the available evidence**

Many at-risk low-income and middle-income countries do not have data on typhoid incidence needed to inform and evaluate vaccine introduction. Even in countries where incidence estimates are available, data are typically geographically and temporally sparse due to the resources necessary to initiate and sustain blood culture surveillance. We found that typhoidal *Salmonella* infection incidence can be estimated from community-based serosurveys using dried blood spots, representing an efficient and scalable approach for generating the typhoid burden data needed to inform typhoid control programs in resource-constrained settings.

Here, we model longitudinal immune responses to HlyE and lipopolysaccharide among patients with enteric fever confirmed by blood culture enrolled from two multi-year, hospital-based, enteric fever surveillance studies: the Surveillance for Enteric Fever in Asia Project (SEAP)^17^ in Bangladesh, Nepal, and Pakistan, and the Severe Typhoid in Africa (SETA)^18^ surveillance study in Ghana. We then used the longitudinal antibody dynamics to estimate typhoidal *Salmonella* seroincidence from cross-sectional population data and compared seroincidence rates to clinical incidence rates in the same catchment areas.^17^, ^18^

## Methods

### Study design and participants

We enrolled patients with blood culture-confirmed typhoidal *Salmonella* infection and collected longitudinal blood samples for up to 2 years. Concurrently, we conducted population-based serosurveys in the catchment areas of each clinical surveillance site. We characterised the dynamics of antibody responses to HlyE and lipopolysaccharide among cases, then applied a model using these kinetics to cross-sectional data to estimate the population-level seroincidence of infection.

All patients with blood culture-confirmed enteric fever enrolled through SEAP were eligible to participate in the ancillary serological study. Patients were enrolled from five hospitals: Dhaka Shishu Hospital (Dhaka, Bangladesh); Kathmandu Medical College and Teaching Hospital (Kathmandu, Nepal); Dhulikhel Hospital (Kavrepalanchok District, Nepal); Aga Khan University Hospital (AKU; Karachi, Pakistan) and Kharadar General Hospital (KGH; Karachi, Pakistan); and a network of laboratory facilities in all three countries between 2016 and 2021. During the enrolment visit, information on demographics, symptom history, and typhoid vaccination status were collected using a structured questionnaire; SEAP enrolment criteria and methods are detailed elsewhere.^17^ For prospective cases, we collected plasma at SEAP enrolment and capillary blood collected on filter paper (ie, dried blood spot) at 28 days, 3 months, 6 months, 12 months, and 18 months post-enrolment. We also enrolled patients retrospectively and collected dried blood spots at enrolment and subsequent scheduled visits following the same follow up intervals. For SETA-Ghana, patients with blood culture-confirmed enteric fever were enrolled from the Agogo Presbyterian Hospital and the Komfo Anokye Teaching Hospital (Agogo, Ghana) between 2016 and 2018. SETA study design and methodology are published elsewhere.^18^ Plasma collected at days 3–7, 28–30, 90, 180, 270, and 360 were included in our analysis. Baseline plasma samples were also collected from 17 patients with invasive non-typhoidal *Salmonella*.

We obtained written informed consent from all eligible participants and the parents or guardians of participants younger than 18 years before collecting blood samples and completing the questionnaire; we obtained written assent from children aged 15–17 years in Bangladesh, Nepal, and Pakistan, and children aged 12–17 years in Ghana. Institutional Review Boards in the USA (Centers for Disease Control and Prevention; Stanford University [39557]; MassGeneral Brigham [2014P002602, 2019P000152, 2013P001965, and 2016P000949]), Bangladesh (Bangladesh Institute of Child Health Ethical Review Committee [01-02-2019]), Nepal (Nepal Health Research Council Ethical Review Board [391/2018]), Pakistan (AKU Ethical Review Committee [2019-0410-4188] and Pakistan National Bioethics Committee [4-87/NBC-341-Amend-revised/19/81]), Korea (International Vaccine Institute IRB), Belgium (Institute of Tropical Medicine Antwerp Institutional Review Board), and Ghana (Komfo Anokye Teaching Hospital, Committee on Human Research, Publication and Ethics) approved the study forms and protocols.

### Randomisation

We enrolled geographically random, population-based samples of individuals aged 0 to 25 years from the catchment areas of SEAP hospitals between 2019 and 2021 (appendix 4 p 4). We randomly selected sampling grid clusters within catchment areas, enumerated all households residing in each cluster, and then randomly selected an age-stratified sample (age 0–4 years, age 5–9 years, age 10–15 years, and age 16–25 years). A description of the randomisation procedure for selecting sampling grids, households, and individuals has been published previously.^19^ Because we sought to enrol a broad representative sample of the population, there were no exclusion criteria other than age and residence in the study catchment area for participating in the cross-sectional survey. For the SETA-Ghana site, we collected plasma from up to four neighbourhood controls, matched by age, sex, and enrolment site between 2016 and 2018 (appendix 4 p 4).

### Serological analysis

Capillary blood samples were collected on TropBioTM filter papers (Cellabs, Brookvale, NSW, Australia), air-dried for 2 h or more at room temperature, and stored with desiccant in individual plastic bags at –20°C until processing. Plasma was stored at –70°C until processing. SEAP study laboratories analysed samples in each country; SETA and north American samples were analysed at Massachusetts General Hospital, Boston, MA, USA. We used kinetic ELISAs to quantify antibody levels in plasma and eluted dried blood spot samples. Details on the ELISA methods are provided in appendix 4 (p 2) along with a comparison of antibody responses measured from dried blood spots and plasma (appendix 4 p 4).

### Statistical analysis

To describe the antibody kinetics of each antigen-isotype following typhoidal *Salmonella* infection, we fitted two-phase models with an exponential rise, peak, and then power-function decay episode.^20^, ^21^ We fitted the models using a Bayesian hierarchical framework obtaining predictive posterior samples using Markov chain Monte Carlo sampling for baseline (y0) and peak antibody responses (y1), time to peak (t1), decay rate (α), and decay shape (r).^21^ The models were run in JAGS version 4.3.0, using the rjags package.^22^ We fitted the longitudinal antibody response models in three age strata (age <5 years, age 5–15 years, and age >15 years) chosen to compare the seroincidence estimates to clinical incidence estimates from the same catchment areas. We reported the median and 95% credible interval (CrI) from each posterior distribution.

When following individuals over time, reinfections are possible and increase in likelihood with longer follow-up. We defined suspected reinfections as the occurrence of a three times or more increase in antibody response in individuals between visits 3 or more months from fever onset for two or more antigen-isotype combinations unless the absolute value of the difference between measurements was less than 1 ELISA unit. The three-times threshold was derived by calculating the median times change from baseline to 28 days among patients with blood culture-confirmed enteric fever or typhoidal *Salmonalla*. Additional details on how we defined reinfections are available in appendix 4 (p 2). Observations including and after the suspected reinfection event were excluded from the longitudinal decay parameter estimation.

To estimate seroincidence, we created a likelihood function for the observed cross-sectional population data based on the longitudinal kinetics following infection.^23^ We assumed that incident infections in the study sample occurred as a Poisson process with rate (λ) and generate maximum likelihood profiles for with each antigen and isotype separately and also jointly estimated by combining their likelihood functions.^24^ We estimated age-stratified incidence rates in the population using the age-specific antibody response parameters. We accounted for two sources of noise in the observed serological responses: measurement noise of the assay (described by the coefficient of variation across replicates) and biological noise (measured as background response to the antigen-isotype among never-exposed, negative controls).^23^ Details on how we specified the measurement and biological noise parameters are provided in appendix 4 (p 2). We used mixed-effect models to adjust the standard errors for clustering by sampling unit for the population-based serosurvey.

We compared our population-based seroincidence estimates to clinical blood culture-based incidence estimates derived from the same catchment area populations. We included both crude incidence (number of culture-confirmed patients divided by the catchment population and observation time) and adjusted incidence estimates (accounting for blood culture sensitivity and the proportion of patients with typhoid-like illness who had blood culture at a surveillance site), following previously described methods.^25^ We estimated the seroincidence rates among children aged 2 to 5 years compared with the clinical incidence estimates reported for this age category. We focused the comparisons among young children, as the seroincidence would reflect recent infections during the same period as the clinical incidence study. Although clinical incidence estimates were available for some catchment areas for children younger than 2 years, we had an insufficient number of young children younger than 2 years in the population samples to estimate seroincidence in this age stratum.

### Role of the funding source

The funder of the study had no role in study design, data collection, data analysis, data interpretation, or writing of the report**.**

## Results

We enrolled a longitudinal cohort of 1420 patients with blood-culture-confirmed enteric fever between May 26, 2016, to Feb 6, 2021 (407 from Bangladesh, 543 from Nepal, 399 from Pakistan, and 71 from Ghana) from the SEAP and SETA studies (table 1). Patients were followed up for a median of 382·0 days after fever onset (IQR 94·0–696·0) and 4126 longitudinal blood samples were collected and analysed (appendix 4 p 5). Median antibody responses at 1 and 6 months after fever onset were comparable across sites and higher than the median values for the serosurvey participants at each location (appendix 4 p 6). Correlation between antibody responses across antigens and isotypes was higher among younger cases and at earlier time points (closer to infection; appendix 4 p 7).

**Table 1:** Demographic, clinical, and sampling characteristics of culture-confirmed enteric fever cases included in the longitudinal kinetic analysis, by country

		**Bangladesh (n=407)**	**Nepal (n=543)**	**Pakistan (n=399)**	**Ghana (n=71)**	**Total (n=1420)**
Age, years		5·4 (3·2–8·0)	20·9 (15·7–26·4)	5·3 (3·0–12·0)	8·0 (6·0–12·0)	9·0 (4·5–19·7)
Age strata						
	<5 years	179 (44·0%)	12 (2·2%)	181 (45·4%)	11 (15·5%)	383 (27·0%)
	5–15 years	227 (55·8%)	127 (23·4%)	152 (38·1%)	52 (73·2%)	558 (39·3%)
	>16 years	1 (0·2%)	404 (74·4%)	66 (16·5%)	8 (11·3%)	479 (33·7%)
	Missing data	0	0	0	0	0
Sex						
	Male	213 (52·3%)	302 (55·6%)	228 (57·1%)	41 (57·7%)	784 (55·2%)
	Female	194 (47·7%)	241 (44·4%)	171 (42·9%)	29 (40·8%)	635 (44·7%)
	Missing data	0	0	0	1 (1·4%)	1 (0·1%)
Number of serum samples collected per participant		2·0 (2·0–3·0)	2·0 (2·0–4·0)	3·0 (2·0–5·0)	4·0 (1·5–5·0)	3·0 (2·0–4·0)
Study enrollment duration, days		184·0 (28·0–540·5)	366·0 (160·0–595·5)	699·0 (367·5–712·0)	270·0 (14·5–365·0)	382·0 (94·0–696·0)
Reported number of days of fever at presentation		4·0 (3·0–6·0)	4·0 (3·0–6·0)	6·0 (4·0–9·0)	6·0 (3·0–7·0)	4·0 (3·0–7·0)
Hospitalised						
	No	326 (80·1%)	372 (68·5%)	210 (52·6%)	31 (43·7%)	939 (66·1%)
	Yes	81 (19·9%)	156 (28·7%)	188 (47·1%)	33 (46·5%)	458 (32·3%)
	Missing data	0	15 (2·8%)	1 (0·3%)	7 (9·9%)	23 (1·6%)
*Salmonella enterica* serovar						
	*S* Paratyphi A	56 (13·8%)	86 (15·8%)	11 (2·8%)	0	153 (10·8%)
	*S* Typhi	351 (86·2%)	457 (84·2%)	388 (97·2%)	71 (100%)	1267 (89·2%)
	Missing data	0	0	0	0	0
Vaccine status						
	Typhoid conjugate vaccine	1 (0·2%)	1 (0·2%)	4 (1·0%)	0	6 (0·4%)
	Other typhoid vaccine	1 (0·2%)	1 (0·2%)	8 (2·0%)	0	10 (0·7%)
	Not vaccinated	405 (99·5%)	541 (99·6%)	378 (94·7%)	71 (100%)	1395 (98·2%)
	Missing data	0	0	9 (2·3%)	0	9 (0·6%)

Data are median (IQR) or n (%)

**Figure 1:**
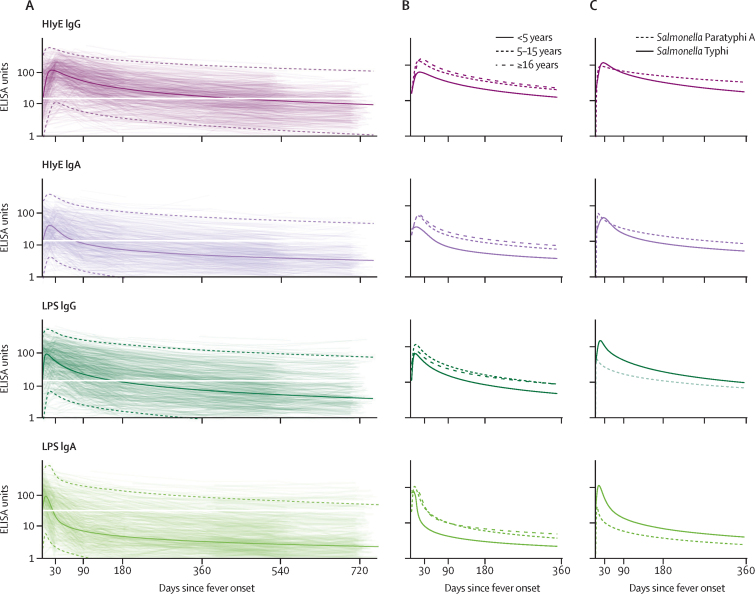
Longitudinal antibody dynamics among patients with blood culture-confirmed enteric fever Longitudinal antibody dynamics were fit to ELISA-measured antibody responses using Bayesian hierarchical models. (A) The light coloured lines are the observed individual antibody concentrations; each line indicates one patient. The dark solid line indicates the median and dotted lines indicate 95% credible intervals for the model-fitted antibody decay concentrations. The white horizontal line marks the median baseline antibody response for each antigen isotype. (B) The median model-fitted antibody trajectories for each age stratum. (C) The median model-fitted antibody trajectories for patients with *Salmonella* Typhi to *Salmonella* Paratyphi A. Ages are restricted to 5–15 years old because patients with *S* Paratyphi A were older than patients with *S* Typhi. HlyE=hemolysin E. LPS=lipopolysaccharide.

37 participants had a three-times or higher increase in antibody responses, meeting the definition of suspected reinfection. The reinfection incidence was 5·2 (95% CI 1·8–8·5) in Bangladesh, 4·9 (4·0–6·8) in Pakistan, 0·7 (0·0–1·6) in Nepal, and 5·6 (0·0–13·3) in Ghana per 100 person-years. The median time to detection of suspected reinfection was 13·8 months (IQR 10·3–18·4) following fever onset. Antibody responses to HlyE and lipopolysaccharide reached peak levels within 3 weeks of fever onset (figure 1A). Peak HlyE antibody responses increased with age. The rate of antibody decay decreased with age across all antigen-isotype combinations (figure 1B; appendix 4 p 15). The overall decay rate for HlyE IgA was slightly faster than for HlyE IgG. The shape parameters for all antigen-isotypes deviate from 1, indicating non-exponential decay (appendix 4 p 15). Median responses remained elevated above baseline levels for 29 months for HlyE IgG, 14 months for lipopolysaccharide IgG, 11 months for HlyE IgA, and 3 months for lipopolysaccharide IgA (figure 1).

**Figure 2:**
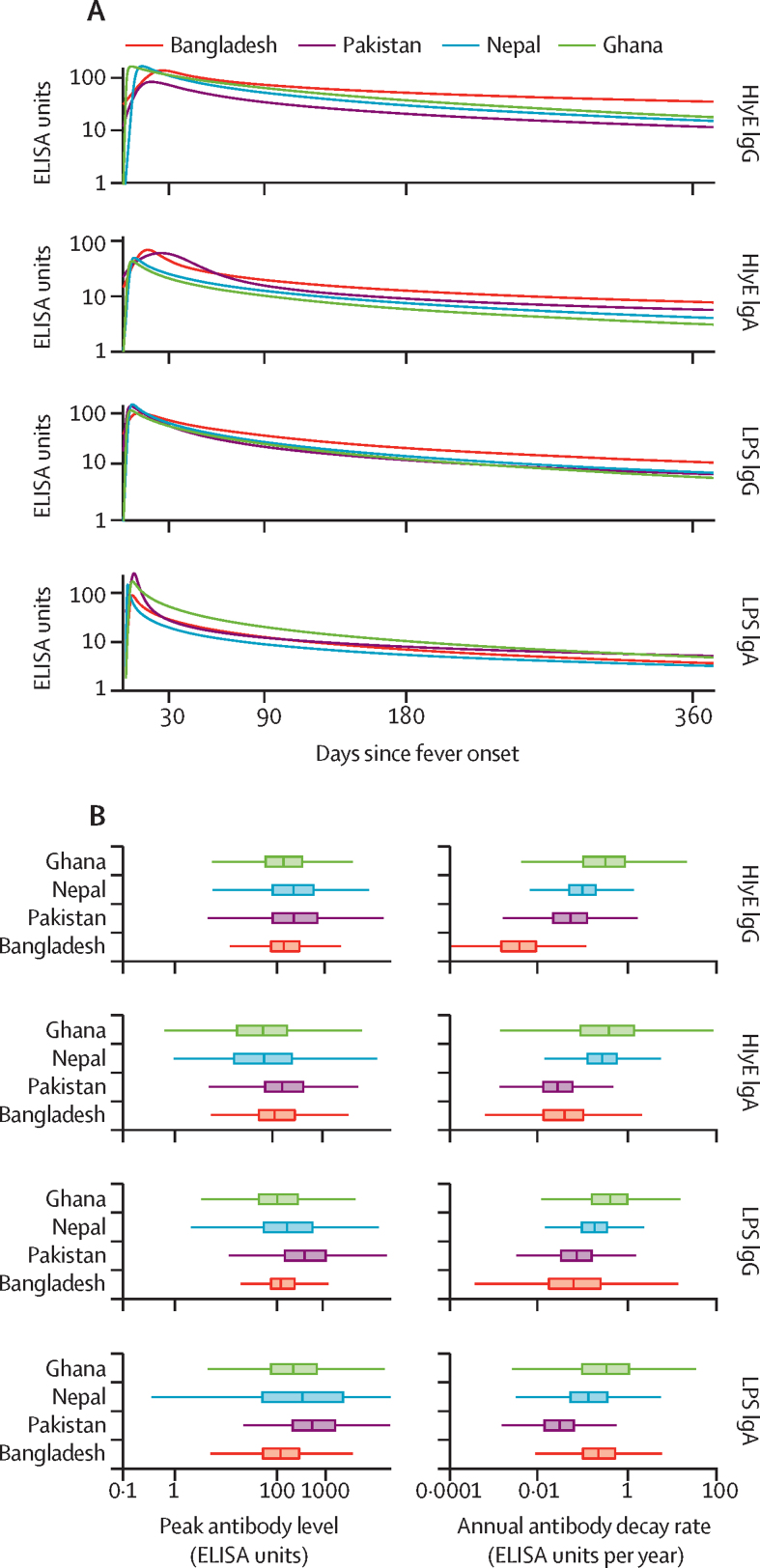
Antibody kinetics, peak response, and decay rate among blood culture-confirmed enteric fever cases by study country All comparisons are among children aged 5 to 15 years to account for the different age-distribution of cases across countries. (A) Median longitudinal antibody decay profiles fit to ELISA-measured antibody responses in each study country. (B) Box plots of model-predicted peak antibody responses and annual antibody decay rates across study countries. HlyE=hemolysin E. LPS=lipopolysaccharide.

The model fitted antibody trajectories were similar across all four study sites (figure 2A). The distributions for peak antibody responses were similar across countries (figure 2B), and the differences between distributions all centred near 0 for all antigen-isotype combinations. There was some inter-country variation in the decay rate, with cases in Bangladesh having a slower decay rate than the other sites (figure 2B).

*S* Paratyphi A was responsible for 153 (10·8%) of 1420 enteric fever cases overall: 56 (13·8%) of 407 in Bangladesh, 86 (15·8%) of 543 in Nepal, 11 (2·8%) of 399 in Pakistan, and none of 71 in Ghana (table 1). Compared with *S* Typhi cases, *S* Paratyphi A cases had similar HlyE IgG and IgA antibody trajectories; however, peak responses for both LPS IgA and IgG were lower (figure 1C). We also compared antibody responses among patients with enteric fever who were hospitalised and not hospitalised, and found similar antibody trajectories for all antigen-isotype combinations (appendix 4 p 8).

In parallel to the longitudinal cohort, we enrolled and analysed dried blood spots from 1808 individuals younger than 26 years in cross-sectional population-based serosurveys: 401 in Dhaka, Bangladesh; 353 in Kathmandu and 481 in Kavrepalanchok, Nepal; 294 from the AKU catchment area, and 200 from the KGH catchment area in Karachi, Pakistan; and 79 in Agogo, Ghana (appendix 4 p 9; table 2). We focused our serosurveys on individuals younger than 26 years, because most patients (whose data was used to parameterise the longitudinal kinetic model) were younger than 26 years (1228 [86·5%] of 1420). Median antibody responses for all antigen-isotypes increased with age and were highest in Dhaka, Bangladesh, and lowest in Kavrepalanchok, Nepal, and Agogo, Ghana (appendix 4 p 10). Compared with the antibody responses observed in cases within 6 months of fever onset, median values for the serosurvey participants were lower for all antigen-isotypes at each site (appendix 4 p 6).

**Table 2:** Demographic and sampling characteristics of the population-based cross-sectional serosurvey participants by country

		**Dhaka, Bangladesh (n=401)**	**Kathmandu, Nepal (n=353)**	**Kavrepalanchok, Nepal (n=481)**	**Aga Khan University Hospital, Karachi, Pakistan (n=294)**	**Kharadar General Hospital, Karachi, Pakistan (n=200)**	**Agogo, Ghana (n=79)**
Age, years		9·2 (4·9–14·0)	12·0 (5·8–17·8)	10·2 (5·1–15·7)	10·0 (4·9–16·0)	8·0 (4·9–13·7)	6·0 (5·0–9·0)
Age strata							
	<5 years	101 (25·2%)	73 (20·7%)	113 (23·5%)	75 (25·5%)	51 (25·5%)	18 (22·8%)
	5–15 years	256 (63·8%)	169 (47·9%)	249 (51·8%)	143 (48·6%)	118 (59·0%)	59 (74·7%)
	16–25 years	44 (11·0%)	111 (31·4%)	119 (24·7%)	76 (25·9%)	31 (15·5%)	2 (2·5%)
	Missing data	0	0	0	0	0	0
Sex							
	Male	183 (45·6%)	184 (52·1%)	248 (51·6%)	135 (45·9%)	97 (48·5%)	47 (59·5%)
	Female	218 (54·4%)	169 (47·9%)	233 (48·4%)	159 (54·1%)	103 (51·5%)	32 (40·5%)
	Missing data	0	0	0	0	0	0
Vaccine status							
	Typhoid conjugate vaccine	13 (3·2%)	0	0	127 (43·2%)	85 (42·5%)	0
	Other typhoid vaccine	17 (4·2%)	3 (0·8%)	1 (0·2%)	3 (1·0%)	1 (0·5%)	0
	Not vaccinated	371 (92·5%)	348 (98·6%)	480 (99·8%)	164 (55·8%)	114 (57·0%)	79 (100%)
	Missing data	0	2 (0·6%)	0	0	0	0

Data are median (IQR) or n (%)

**Figure 3:**
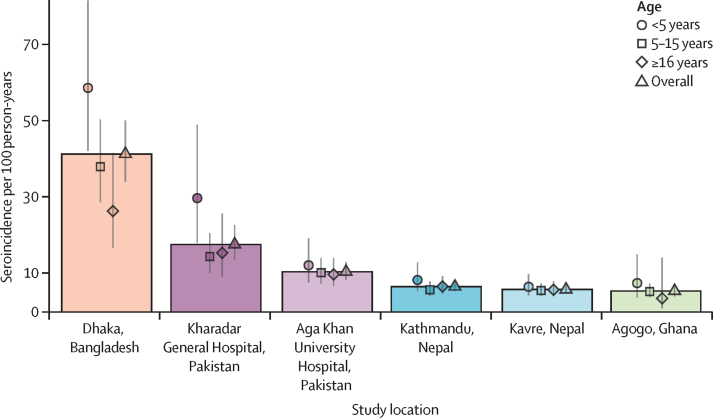
Estimated seroincidence of typhoidal *Salmonella* by study community and age group Age groups are denoted by point shapes for the median, with lines indicating the 95% CI. Boxes reflect the height of the median estimate for the overall population-based serosurvey.

We used only HlyE IgG and IgA to estimate seroincidence because lipopolysaccharide antibody responses were lower among *S* Paratyphi A cases (figure 1C) and we identified elevated lipopolysaccharide antibody responses among patients with invasive non-typhoidal Salmonellosis (appendix 4 p 11),^21^, ^22^ suggesting possible cross-reactivity. The seroincidence rate of enteric fever per 100 person-years was highest in Dhaka, Bangladesh (﻿41·2 [95% CI 34·0–50·1]), followed by Karachi, Pakistan (﻿17·6 [13·7–22·6] in KGH; 10·5 [8·5–13·0] in AKU), Nepal (﻿6·6 [5·4–8·1] in Kathmandu; 5·8 [4·8–7·1] in Kavrepalanchok), and Agogo, Ghana (5·5 [4·1–7·35]; figure 3). In Dhaka, Agogo, and the KGH and AKU catchment areas, the seroincidence was higher in the youngest age group (aged <5 years). In Kathmandu and Kavrepalanchok the seroincidence was similar across age strata. Seroincidence estimates using anti-HlyE and anti-lipopolysaccharide IgA and IgG are presented in appendix 4 (p 16). The differences in incidence estimates using overall versus country-specific longitudinal parameters (peak, decay rate, and decay shape) are presented in appendix 4 (p 12).

**Figure 4:**
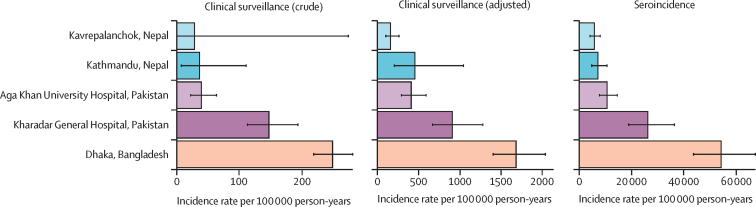
Comparison of estimates for crude and adjusted clinical enteric fever incidence with typhoidal *Salmonella* seroincidence Crude incidence reflects the number of culture-confirmed *Salmonella* Typhi and *Salmonella* Paratyphi A cases divided by the catchment population and time. Adjusted incidence accounts for imperfect sensitivity of blood culture and the proportion of acute febrile illnesses captured by the surveillance system. The horizontal axis indicates incidence, and scale differs for type of estimate. Estimates are shown for children younger than 5 years, for the serological estimates to coincide with the period of clinical surveillance.

Among children aged 2–4 years, the seroincidence estimates followed the same rank order as the clinical incidence estimates for each catchment area. Dhaka, Bangladesh, had the highest seroincidence, followed by KGH and AKU in Karachi, Pakistan, and then Kathmandu and Kavrepalanchok, in Nepal (figure 4). Seroincidence estimates were between 16 and 32 times higher than care-seeking adjusted clinical incidence rates (incidence ratio: 32·3 in Bangladesh; 28·9 in KGH; 26·1 in AKU; 36·7 in Kavrepalanchok; and 15·9 in Kathmandu).

In a sub-analysis of samples from patients who were blood culture-positive for enteric fever in Nepal, we found that after a modest rise, anti-Vi antibody responses plateaued with no subsequent decay (rate=0; appendix pp 13, 15). Median anti-Vi IgG levels remained elevated above baseline for at least 32 months (appendix p 13). The ratio of 28-day responses among patients compared with the population-level mean for each antigen-isotype was lowest for anti-Vi IgG (median 1·5 [IQR 1·0–2·1]) and highest for anti-HlyE IgG (30·9 [16·1–54·6]; appendix p 13). Anti-Vi IgG responses 28 days post-infection were elevated 1·5 times above the population mean; anti-HlyE IgG responses were 30 times higher than the population means.

## Discussion

We describe an approach to estimate typhoidal *Salmonella* infection seroincidence from cross-sectional serosurveys using finger-stick dried blood spots. Leveraging longitudinal cohorts of patients with enteric fever in four countries, we found IgA and IgG responses to HlyE and lipopolysaccharide were markedly increased for many months following infection, and these elevations were consistent across populations with varying infection pressures. We then used HlyE IgA and IgG to estimate the seroincidence of typhoidal *Salmonella* infection in the catchment areas of six study sites across four countries with prospective blood culture-based surveillance, finding the rank order of seroincidence estimates tracked with clinical enteric fever incidence estimates.

Unlike traditional seroepidemiological methods that rely on a cutoff, we used quantitative, longitudinal antibody responses, integrating IgA and IgG isotype data, to generate population-level seroincidence estimates. A strength of this approach is that we incorporated several types of uncertainty: measurement error, biological noise, and inter-individual variation in antibody responses. Although the antibody responses showed considerable individual variation among patients, we found the median fitted peak antibody response was consistent across the four countries. There was some variation in the decay rates across countries, with Bangladesh having the slowest decay rate and Ghana having the fastest. We propose two potential explanations for this difference. The first is that reinfections are more common in settings with a higher force of infection. We classified suspected reinfections in the longitudinal case data; however, some reinfections might not have been identified. The second is that in settings with a high incidence of enteric fever, individuals with typhoid are more likely to be frequently exposed, and secondary antibody responses might wane more slowly.

Previous typhoid seroepidemiological studies have used IgG responses to the Vi polysaccharide capsule. We found the ratio of 28-day antibody responses among cases compared with the population serosurvey participants was lowest for anti-Vi IgG and highest for anti-HlyE IgG, implying anti-Vi IgG does not accurately distinguish acute cases from previous exposures. Furthermore, we found no increase in serological responses to Vi by age, echoing an earlier study from Kathmandu.^26^ A study from Fiji found anti-Vi IgG responses increase with age but identified no differences in age-response patterns between high-incidence and low-incidence communities.^8^ A 2021 study from Malawi, Nepal, and Bangladesh used a change in Vi IgG response between two longitudinal samples to mark an incident infection and found a similar order of magnitude to the seroincidence estimates presented here.^27^ However, the use of Vi in WHO-recommended vaccines will preclude distinguishing natural infections from vaccine-derived immunity. Together, these circumstances underscore the importance of using alternative antigens in assessing enteric fever seroincidence.

The seroincidence estimates for all catchment areas were substantially higher than population-based clinical incidence estimates, even after adjusting for care-seeking and blood culture sensitivity. A high incidence of clinical enteric fever is defined as more than 100 cases per 100 000 person-years.^28^ We estimated seroincidence of more than 4000 per 100 000 person-years in all study areas, with rate ratios of seroincidence to clinical incidence ranging from 16 to 32. This estimate implies a substantial incidence of asymptomatic or paucisymptomatic infections, as observed with other infections.^6^, ^29^, ^30^, ^31^, ^32^ One benefit of community-based seroincidence estimates is their robustness to differential care-seeking behaviour, health-care access, and cultural differences in reporting illness, which might enable less biased estimates of the force of infection in a community.

In south Asia, 10–20% of enteric fever cases are caused by *S* Paratyphi A.^17^ We found antibody responses for *S* Typhi lipopolysaccharide were lower among patients with *S* Paratyphi A infection than among patients with *S* Typhi infection, which we expected given that *S* Paratyphi A only shares the O12 antigen with *S* Typhi (*S* Typhi has O9 and O12 antigens). Additionally, we observed elevated anti-lipopolysaccharide antibody responses among patients with invasive non-typhoidal *Salmonella* from Ghana*.* These cross-reactive responses are probably due to the shared O12 antigen, a trisaccharide repeat backbone identical among *Salmonella* groups A, B, and D.^16^, ^33^ As such, we used HlyE antibody responses, which did not differ between *S* Typhi and *S* Paratyphi A, for our primary seroincidence estimates. Our earlier studies have demonstrated high specificity of anti-HlyE antibody responses for typhoid or paratyphoid diagnosis, compared with other bacterial infections.^11^, ^34^ We are working to identify *S* Typhi-specific or *S* Paratyphi-specific immune responses to enable serovar-specific seroincidence estimation.

The results of this study should be interpreted within the context of several limitations. First, we incorporated age-dependence in antibody responses among patients with enteric fever by fitting separate kinetics models to each age stratum. Methods to formally account for age dependence in the longitudinal decay curves require further development. Second, we focused on our serosurveys on individuals younger than 26 years. Characterising longitudinal antibody responses in older individuals would be needed to calculate seroincidence in older ages and would be of value given the consideration for catch-up typhoid conjugate vaccine campaigns in individuals up to age 45 years. Although the clinical incidence of typhoidal *Salmonella* is higher among older ages in the SEAP study sites in Nepal, we observed a stable seroincidence rate across ages. Possible explanations for this observation are that the ratio between seroincidence and clinical infections might differ by age or that seroincidene estimates might be less reliable at older ages due to multiple exposures and differential waning. The seroincidence estimates reflected the age pattern of clinical incidence for the Bangladesh (Dhaka) and Pakistan (KGH) study sites. Third, we assumed the antibody kinetics were similar for all patients, but it is possible that asymptomatic individuals have different antibody kinetics. We found antibody kinetics did not differ between hospitalised and non-hospitalised patients with enteric fever, suggesting that antibody responses are not dependent on clinical disease severity. However, we could not determine whether individuals who have asymptomatic infections or who receive care at lower acuity facilities have similar antibody responses. Our method would underestimate seroincidence if the peak antibody responses are lower and the decay more rapid for asymptomatic cases. Fourth, in regions with high transmission intensity, individuals might be frequently re-exposed, and the shape and parameters of antibody kinetics from secondary and tertiary exposures will probably differ from primary infections.^35^ Longitudinal studies with serial blood culture are needed to accurately describe the kinetics of true reinfections. Fifth, long-term carriage of typhoidal *Salmonella* might alter the longitudinal serological responses, though anti-HlyE antibody responses were not identified on an immunoscreen among carriers.^36^ Sixth, although we know typhoid transmission is seasonal in some environments, our modelling approach assumes a constant force of infection throughout the year and therefore averages the incidence rate across the year. Finally, although serosurveys might provide an efficient means for incidence estimation in resource-constrained settings, they do not obviate the need for blood culture surveillance, which is the only method for monitoring antimicrobial resistance—a serious and growing threat to the effective treatment of typhoid.

WHO recommends that countries with high typhoid incidence introduce typhoid conjugate vaccines in national programmes, creating an urgent need for typhoidal *Salmonella* incidence data. Clinical surveillance for enteric fever is limited to settings with facilities equipped to perform blood cultures; even when available, incidence estimates might be highly sensitive to biases in care-seeking behaviours, antibiotic use, and variable diagnostic sensitivity.^25^ We describe a serosurveillance approach that efficiently generates population-level typhoidal *Salmonella* incidence estimates. We found that sample sizes of 200 to 400 individuals per age strata were sufficient to consistently estimate incidence depending on the burden of typhoid in the population, with higher burden settings requiring smaller sample sizes. This approach has the potential to expand the geographical scope of typhoid surveillance, generate much-needed subnational data on its burden, and yield incidence estimates that are comparable across geographical regions and time.

## Data sharing

All analysis code is available at https://github.com/kaiemjoy/TyphoidSeroIncidence.git.

## Supplementary Materials

Nepali translation of the abstract

Bengali translation of the abstract

Urdu translation of the abstract
