# Correction to *Lancet Microbe* 2022; 3: e578–87

**DOI:** 10.1016/S2666-5247(23)00037-X

**Published:** 2023-03

*Aiemjoy K, Seidman JC, Saha S, et al. Estimating typhoid incidence from community-based serosurveys: a multicohort study.* Lancet Microbe *2022;* 3: *e578–87*—In this Article, the fourth sentence of the figure 4 legend should have read “Estimates are shown for children aged 2–4 years for the serological estimates to coincide with the period of clinical surveillance”. This correction has been made to the online version as of Feb 6, 2023.

