# Supplementary material for: Estimating typhoid incidence from community-based serosurveys: a multicohort study
Source: Lancet Microbe. 2022 Aug;3(8):e578–87. doi: 10.1016/S2666-5247(22)00114-8 (PMC9329131; doi:10.1016/S2666-5247(22)00114-8)
Supplement: Nepali translation of the abstract [file mmc1.pdf]

THE LANCET
Microbe

This translation in Nepali was submitted by the authors and we reproduce it as supplied. It has not been peer reviewed. *The Lancet* editorial processes have only been applied to the original in English, which should serve as reference for this manuscript.

नेपालीमा यो अनुवाद लेखकहरूद्वारा पेश गरिएको हो र हामीले यसलाई आपूर्ति गरे अनुसार उर्ताएका छौं। यसलाइ सहकर्मी समीक्षा गरिएको छैन। ल्यान्सेटको सम्पादकीय प्रक्रियाहरू मूल अंग्रेजीमा मात्र लागू गरिएको छ, जसले यस पाण्डुलिपिको निर्देश ग्रन्थको रूपमा काम गर्नुपर्छ।

Supplement to: Aiemjoy K, Seidman J C, Saha S, et al. Estimating typhoid incidence from community-based serosurveys: a multicohort study. *Lancet Microbe* 2022; published online June 21. https://doi.org/10.1016/S2666-5247(22)00114-8.

सार

पृष्ठभूमि : रगत कल्चरको निगरानी (surveillance) उपलब्ध नभएका क्षेत्रहरूमा टाइफाइडल सल्मोनेला (Typhodial Salmonellae) नामक एक संक्रामक जीवाणुको संक्रमणबाट हुने टाइफाइड ज्वरोको इन्सिडेन्स धेरै हदसम्म अज्ञात अवस्थामा छ। यो अध्ययनको लक्ष्य यदि टाइफाइडल सल्मोनेलोको नयाँ सेरोलोजिक मार्करहरूले जनसंख्या-स्तरको इन्सिडेन्सको विश्वसनीय अनुमान गर्न सक्ने मूल्याङ्कन गर्नु थियो।

विधिहरू : हामीले बंगलादेश, नेपाल, पाकिस्तान, र घानामा गरिएको निगरानी अध्ययनमा सहभागी भएका रगत कल्चरबाट प्रमाणित टाइफाइड ज्वरोका बिरामीहरूको पटक-पटक रगत नमुना सङ्कलन गर्‍यौं र प्रत्येक अध्ययन स्थलका लागि तोकिएका क्षेत्रहरूमा Cross-sectional सेरोसर्वेक्षणहरू गर्‍यौं। हामीले ELISA विधि प्रयोग गरेर Hemolysin E (HlyE) र *S.typhi* lipopolysaccharide (LPS) बिरुद्धको IgA र IgG एन्टिबडी प्रतिक्रियाको मात्रा मापन गर्‍यौं। हामीले टाइफाइड ज्वरोका बिरामीहरूमा longitudinal एन्टिबडी प्रतिक्रियाहरू अनुमान गर्न Bayesian hierarchical models to fit two-phase power-function decay models; र cross-sectional सेरोसर्वेक्षणबाट जनसंख्या-स्तरको इन्सिडेन्स अनुमान गर्न joint distributions of peak antibody titers and decay rate को प्रयोग गर्‍यौं।

निष्कर्ष : सबै antigen-isotypes का longitudinal antibody kinetic हरू सबै देशहरूमा समान देखिए; साथै, ती स्वास्थ्य गम्भीरताका आधारमा भिन्न देखिएनन्। पाँच वर्ष मुनिका बच्चाहरूमा टाइफाइड साल्मोनेला संक्रमणको सेरो-इन्सिडेन्स बंगलादेशको ढाकामा ५८.५ प्रति १०० व्यक्ति-वर्ष (९५% CI : ४२.१ - ८१.४) देखि नेपालको काभ्रेपलाञ्चोकमा ६.६ प्रति १०० व्यक्ति-वर्ष (९५% CI : ४.३-९.९) सम्म छ, र क्लिनिकल इन्सिडेन्सको अनुमानको श्रेणी-क्रम पालना गर्दछ।

व्याख्या : टाइफाइडल साल्मोनेला निगरानीको भौगोलिक दायरा विस्तार गर्न तथा इन्सिडेन्स उत्पादन गरी भौगोलिक क्षेत्र र समयअनुसार तुलनात्मक अनुमान प्रस्तुत गर्न यहाँ वर्णन गरिएको प्रक्रियाले सम्भाव्यता राख्दछ।
