# Supplementary material for: Estimating typhoid incidence from community-based serosurveys: a multicohort study
Source: Lancet Microbe. 2022 Aug;3(8):e578–87. doi: 10.1016/S2666-5247(22)00114-8 (PMC9329131; doi:10.1016/S2666-5247(22)00114-8)
Supplement: Bengali translation of the abstract [file mmc2.pdf]

THE LANCET
Microbe

Supplementary appendix

This translation in Bengali was submitted by the authors and we reproduce it as supplied. It has not been peer reviewed. *The Lancet*'s editorial processes have only been applied to the original in English, which should serve as reference for this manuscript.

ঘোষণা

এই বঙ্গানুবাদটি নিবন্ধের লেখকের দ্বারা লিখিত, এবং হুবহু সেভাবে এখানে প্রদান করা হয়েছে। এটি পিয়ার রিভিউড (পর্যালোচিত) নয়। দি ল্যান্সেটের সম্পাদনা প্রক্রিয়াসমূহ কেবল মূল ইংরেজী নিবন্ধটির ক্ষেত্রে প্রযোজ্য, যেটি এই পান্ডুলিপির ক্ষেত্রে সূত্র হিসেবে বিবেচিত হবে।

Supplement to: Aiemjoy K, Seidman J C, Saha S, et al. Estimating typhoid incidence from community-based serosurveys: a multicohort study. *Lancet Microbe* 2022; published online June 21. https://doi.org/10.1016/S2666-5247(22)00114-8.

পটভূমি:

টাইফয়েড জ্বর, সালমোনেলা টাইফি ব্যাকটেরিয়া দ্বারা সৃষ্ট একটি সংক্রামক ব্যাধি। কিন্তু ভালো ল্যাবরেটরী ব্যবস্থাসহ রক্তের কালচার ভিত্তিক নজরদারী না থাকলে টাইফয়েডের জীবাণু সুনির্দিষ্টভাবে শনাক্ত করা যায় না। আমরা টাইফয়েড জ্বর নির্ণয়ের জন্য নতুন কিছু সেরোলজিক মার্কার (রক্তরস-ভিত্তিক পরীক্ষা) চিহ্নিত করেছি। তবে এই চিহ্নিত মার্কারগুলো জনসংখ্যা জরিপে নির্ভরযোগ্যভাবে ব্যবহার করা যাবে কিনা তা এখনও জানা যায়নি।

পদ্ধতি:

আমরা বাংলাদেশ, নেপাল, পাকিস্তান এবং ঘানার সূনির্দিষ্ট এলাকার নিশ্চিত টাইফয়েড রোগীদের থেকে যদৃচ্ছা (cross-sectional) নমুনা সংগ্রহ করেছি। তদুপরি আমরা প্রতিটি দেশের একটি সুনির্দিষ্ট এলাকার জনসংখ্যার মধ্যে সেরোলজিক জরিপ পরিচালনা করেছি।

সংগৃহীত এই রক্তের নমুনাগুলোতে ELISA পরীক্ষার মাধ্যমে Haemolysis E (HlyE) এবং সালমোনেলা টাইফির Lipopolysaccharide (LPS) এর বিরুদ্ধে IgA এবং IgG এর এন্টিবডি পারিমাপ করেছি।

Bayesian hierarchical মডেল ব্যবহার করে, রোগীদের এই এন্টিবডির সর্বোচ্চ এবং সর্বনিম্ন পরিমান নির্ণয় করে আমরা নির্দিষ্ট জনগোষ্ঠীতে টাইফয়েডের ব্যপকতা সম্পর্কে জানতে পারবো।

ফলাফল:

১২ থেকে ১৮ মাস ব্যাপী এই গবেষণায় টাইফয়েডের অ্যান্টিজেন-আইসোটাইপের অ্যান্টিবডিগুলো ভিন্ন ভিন্ন এবং সকল দেশের জনগোষ্ঠীদের মধ্যে রোগের তীব্রতার কোন পরিবর্তন লক্ষ্য করা যায়নি। ৫ বছরের কম বয়সী শিশুদের মধ্যে টাইফয়ডের সেরোলজিক ব্যাপকতা (রক্তরস পরীক্ষার মাধ্যমে নির্ণীত), বাংলাদেশে প্রতি ১০০ জনে বছরে ৫৮.৫ (৯৫% কনফিডেন্স ইনটারভাল ৪২.১- ৮১.৪) থেকে কাভরেপালনচোক, নেপালে ৬.৬ (৯৫% কনফিডেন্স ইনটারভালঃ ৪.৩- ৯.৯) পাওয়া গিয়েছে। এসব এলাকায় টাইফয়েড জ্বরে আক্রান্ত শিশুর সংখ্যাও আমাদের এই ফলাফলকে সমর্থন করেছে।

ব্যাখ্যা:

বিশ্বের বিভিন্ন ভৌগলিক এলাকাতে টাইফয়েড জ্বরের সংক্রমনের ব্যপকতা নির্ণয়ের ক্ষেত্রে আমাদের বর্ণিত পদ্ধতিটি একটি সম্ভাবনাময় পদ্ধতি, যেটা ব্যবহার করে বিভিন্ন অঞ্চলের মধ্যে টাইফয়েডের প্রাদুর্ভাব তুলনা করা যেতে পারে।
