# Supplementary material for: Estimating typhoid incidence from community-based serosurveys: a multicohort study
Source: Lancet Microbe. 2022 Aug;3(8):e578–87. doi: 10.1016/S2666-5247(22)00114-8 (PMC9329131; doi:10.1016/S2666-5247(22)00114-8)
Supplement: Urdu translation of the abstract [file mmc3.pdf]

THE LANCET
Microbe

Supplementary appendix

This translation in Urdu was submitted by the authors and we reproduce it as supplied. It has not been peer reviewed. *The Lancet*'s editorial processes have only been applied to the original in English, which should serve as reference for this manuscript.

اردو میں یہ ترجمہ مصنفین کے ذریعہ پیش کیا گیا تھا اور ہم اس کی ہو بہو دوبارہ تخلیق کرتے ہیں۔ اس کا ہم مرتبہ ماہروں کے ذریعہ جائزہ نہیں لیا گیا ہے۔ *لانسیٹ* کے ادارتی عمل کا اطلاق صرف انگریزی کے اصل پر ہوا ہے، جو اس نسخے کے حوالہ کے طور پر کام کرے گا

Supplement to: Aiemjoy K, Seidman J C, Saha S, et al. Estimating typhoid incidence from community-based serosurveys: a multicohort study. *Lancet Microbe* 2022; published online June 21. https://doi.org/10.1016/S2666-5247(22)00114-8.

خلاصہ

پس منظر

آنتوں کا بخاریا ٹائفائیڈ ایک مہلک جراثیم سے ہونے والی بیماری ہے جو کہ ان علاقوں میں بہت حد تک غیر معروف ہے جہاں خون میں انفیکشن پھیلانے والے جرثوموں کی تشخیص مشکل ہے۔ نئے سیرولوجک مارکر(خون میں موجود اینٹی باڈیز) سے ٹائفائیڈ کی درست تشخیص کے لیے کارآمد ہیں۔ مگر ہم یہ نہیں جانتے کہ آبادی کی سطح پر ان سیرولوجیکل مارکر کا استعمال کتنا معتبر ہو سکتا ہے۔

تحقیقاتی طریقہ کار

ہم نے پاکستان، نیپال، بنگلہ دیش اور گھانا میں جاری مطالعے میں مندرج ان لوگوں سے ترتیب وار خون کے نمونے لیے جن کے خون میں ٹائفائیڈ کے جرثوموں کی موجودگی ثابت ہو چکی تھی۔ ہم نے ان ملکوں میں جن جگہوں سے خون کے نمونے حاصل کیے تھے، وہاں سے آبادی کی سطح پرserosurvey کیا ۔اس کے بعد ہم نے خون کے نمونوں کو ELISA کی مدد سے سالمونیلا ٹائیفی *S. Typhi* جرثومے کے دو اینٹی جنز (antigens) ہیمولائسن-ای (Hemolysin-E) اور لیپوپولی سیکرائیڈ (Lipopolysaccharide) کے خلاف موجود IgA اور IgG اینٹی باڈیز کی پیمائش کی۔ ہم نے جدید تجزیئے کے Bayesian Hierarchical Model کا استعمال دو حصوں پر مبنی پاور فنکشن کے decay models کو آنتوں کے بخار کے کیسوں میں وقتا فوقتا اینٹی باڈی ردعمل جانچنے کے لیے کیا اور انفرادی سطح پر آبادی کی بنیاد پر ہونے والے واقعات کی شرح کا اندازہ لگانے کے لیے انتہا کے اینٹی باڈی ٹائٹرز اور بکھرنے کی شرح کا استعمال کیا۔

نتائج

تمام اینٹیجن-آئسوٹائپس کے لیے ترتیب وار اینٹی باڈی کائینیٹکس (kinetics) تمام ممالک میں یکساں تھے اور مر ض کی شدت کے لحاظ سے مختلف نہیں تھے۔ ڈھاکہ، بنگلہ دیش میں 5 سال سے کم عمر کے بچوں میں خون میں اینٹی باڈیز کی موجودگی کے لحاظ سے سالمونیلا ٹائیفی انفیکشن کی شرح 58.5 فی 100 شخصی سال (95% CI: 42.1- 81.4) سے کوری پالن چوک، نیپال میں 6.6 (95% CI: 4.3-9.9) کے درمیان تھی اور طبی واقعات کے تخمینے کے مطابق ایک ہی درجہ بندی میں تھی۔

نتائج کی توضیح

ہمارا یہاں بیان کردہ طریقہ کار اس قابل ہے کہ اسے دیگر مختلف جغرافیائی علاقوں میں ٹائیفائڈ کے انفیکشن کی نگرانی اور ان آبادیوں میں اس انفیکشن کا تخمینہ کرنے کے لیے مختلف جغرافیائی علاقوں اور وقت کی تفریق کے بغیر استعمال کیا جا سکتا ہے۔
